# Vitamin D Supplementation and Nordic Walking Training Decreases Serum Homocysteine and Ferritin in Elderly Women

**DOI:** 10.3390/ijerph15102064

**Published:** 2018-09-20

**Authors:** Anna Walentukiewicz, Anna Lysak-Radomska, Joanna Jaworska, Krzysztof Prusik, Katarzyna Prusik, Jakub Antoni Kortas, Marcin Lipiński, Anna Babinska, Jedrzej Antosiewicz, Ewa Ziemann

**Affiliations:** 1 Faculty of Rehabilitation and Kinesiology, Department of Health Promotion and Posturology, Gdansk University of Physical Education and Sport, K. Gorskiego 1, 80-336 Gdansk, Poland; annawalentukiewicz@wp.pl; 2 Faculty of Rehabilitation and Kinesiology, Department of Physiotherapy, Gdansk University of Physical Education and Sport, K. Gorskiego 1, 80-336 Gdansk, Poland; anna.lysakradomska@gmail.com; 3 Faculty of Rehabilitation and Kinesiology, Department of Physiology and Pharmacology, Gdansk University of Physical Education and Sport, Gorskiego 1, 80-336 Gdansk, Poland; jaworska.joanna12@gmail.com; 4 Faculty of Tourism and Recreation, Department of Health Promotion, Gdańsk, Gdansk University of Physical Education and Sport, K. Gorskiego 1, 80-336 Gdansk, Poland; krzysztof.prusik@awf.gda.pl (K.P.); prusikkatarzyna@gmail.com (K.P.); jakubantonikortas@gmail.com (J.A.K.); 5 Department of Biochemistry, Medical University of Gdańsk, M. Skłodowskiej-Curie 3, 80-001 Gdansk; Poland; marlip@gumed.edu.pl; 6 Department of Endocrinology and Internal Medicine, Medical University of Gdańsk, M. Sklodowskiej-Curie 3, 80-001 Gdansk, Poland; a.mail@wp.pl; 7 Department of Bioenergetics and Physiology of Exercise, Medical University of Gdańsk, M. Sklodowskiej-Curie 3, 80-001 Gdansk, Poland; jant@gumed.edu.pl

**Keywords:** physical training, methionine, cysteine, brain-derived neurotrophic factor (BDNF)

## Abstract

The aim of the study was to verify if coupling 12 weeks of vitamin D supplementation and Nordic walking training favoured lowering the homocysteine (Hcy) level. Ninety-four elderly women were divided into three groups: Nordic walking (NW), supplemented (SG) and control (CG). The NW and SG groups received a weekly dose of 28,000 IU of vitamin D3. A blood analysis was performed at baseline, 1h after the first training session and at the end of the experiment. The amino acid profile (methionine and cysteine) and homocysteine concentration were determined. Additionally, the concentration of myokine was assessed. The first session of NW training reduced serum homocysteine, particularly among women with baseline homocysteine above 10 µmol·L^−1^: 12.37 ± 2.75 vs. 10.95 ± 3.94 µmol·L^−1^ (*p* = 0.05). These changes were accompanied by shifts in the cysteine (*p* = 0.09) and methionine (*p* = 0.01) concentration, regardless of the Hcy concentration. Twelve weeks of training significantly decreased the homocysteine (9.91 ± 2.78, vs. 8.90 ± 3.14 µmol·L^−1^, *p* = 0.05) and ferritin (94.23 ± 62.49 vs. 73.15 ± 47.04 ng·mL^−1^, *p* = 0.05) concentrations in whole NW group. Also, in the NW group, ferritin correlated with the glucose level (r = 0.51, *p* = 0.00). No changes in the myokine levels were observed after the intervention. Only the brain-derived neurotrophic factor dropped in the NW (42.74 ± 19.92 vs. 31.93 ± 15.91 ng·mL^−1^, *p* = 0.01) and SG (37.75 ± 8.08 vs. 16.94 ± 12.78 ng·mL^−1^, *p* = 0.00) groups. This study presents a parallel decrease of homocysteine and ferritin in response to regular training supported by vitamin D supplementation.

## 1. Introduction

An elevated level of homocysteine (Hcy) is an independent and well-known predictor of civilization diseases. Hcy is defined as vasotoxin and neurotoxin [1,2]. Many independent studies have confirmed the active participation of this sulfhydryl-containing amino acid, which is an intermediate product in the normal biosynthesis of the amino acids methionine and cysteine, in the induction of the arteriosclerosis process [3] and cardiovascular incidents, including strokes [4]. It has been shown that Hcy levels could be connected with neurodegenerative changes in the central nervous system, as an independent risk factor for conditions including Parkinson and Alzheimer diseases, and accompanying cognitive impairment and dementia. Additionally, the serum homocysteine level is an important predictor of mineral bone density, especially in the case of postmenopausal women with diagnosed osteoporosis [5]. It has been reported that Hcy affects the bone remodeling processes, vascular blood flow, and the progression of bone diseases [6], including osteoporotic fractures [7]. An elevated level of Hcy is commonly observed in the elderly together with other pro-inflammatory indicators like an elevated level of body iron stores manifested by an increased level of serum ferritin [8,9]. Ferritin is built from two subunits, ferritin H and L, and is present in all human cells. The function of ferritin is to store iron and to protect cells from its toxicity [10]. Regular exercise has been shown to reduce both serum Hcy and ferritin [2,11,12], however, there have been no studies where the interdependence of these two factors has been evaluated. A decrease in serum ferritin is considered to be related to a decrease in body iron stores. Ferritin H has been shown to stimulate folate catabolism [10]. In addition, the labile iron pool has been shown to increase Hcy formation from methionine [13]. Therefore, we have hypothesized that regular exercise would induce a decrease in the body iron stores, manifested by the diminishing of serum ferritin and accompanied by a drop in serum Hcy.

Recently published papers have demonstrated that the effectiveness of a popular training program, Nordic walking, for reducing pro-inflammatory indictors was modulated by the basic vitamin D concentration [14] and by the training experience itself [15]. This has a particular meaning given the commonly observed deficiency of vitamin D [16]. At the same time, an investigation into different doses used for vitamin D supplementation and their influence has reported a beneficial effect when following the recommendations of the British Nutrition Foundation, the American Geriatrics Society, and the World Health Organization for people aged 65 years or above [17].

On the other hand, the results of a large meta-analysis have also demonstrated that aerobic intervention such as walking, cycle ergometer, or step climbing did not reduce the Hcy concentration. In contrast, regular resistance training led to a decrease in the plasma homocysteine concentration [18]. In fact, exercise variables such as duration, volume, and intensity can be considered as factors regulating the different responses of Hcy formation to acute and chronic exercise [18]. As the skeletal muscle is the largest organ in the body, the fact that it can be a source of anti-inflammatory cytokines [19] has become very important and helpful in understanding the anti-inflammatory effects of exercise. In particular, data published lately have presented and justified a connection between myokines, like irisin and interleukin 6 (IL-6), and the brain-derived neurotrophic factor (BDNF) [20]. Therefore, the aim of the current study was to verify if the coupling of vitamin D supplementation and NW training favored lower homocysteine levels effectively as opposed to training applied in the absence of the additional treatment. We have hypothesized that regular NW training would reduce body iron stores and that this effect will be accompanied by a decrease in the Hcy concentration.

## 2. Materials and Methods 

### 2.1. Study Design

**Figure 1:**
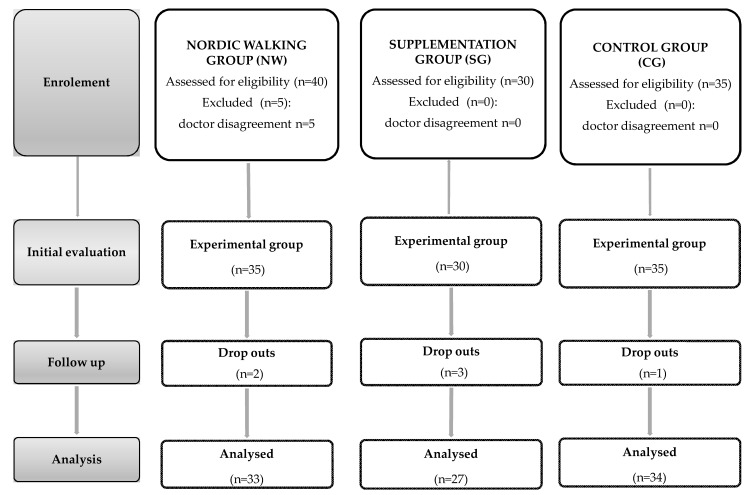
Study design.

A group of 94 women took part in the experiment, which was held at the University of Physical Education and Sport in Gdansk. Subjects were selected according to their age, 60 years old and older (68 ± 5.12 years old). A medical check-up was requested from all participants prior to the experiment. The exclusion criteria for participation included: Uncontrolled hypertension (diastolic blood pressure over 100 mmHg), a history of cardio-respiratory disorders, cardiac arrhythmia, and orthopedic problems. Participants were asked not to change their diet and lifestyle during the 12 weeks of the experiment as well as to refrain from introducing any supplementation treatments. Qualified individuals were randomly assigned to one of the three groups: The Nordic walking group, NW (n = 33); the supplemented group, SG (n = 27); or the control group, CG (n = 34). In order to avoid discrepancies in the level of education between the participants, group allocation was made according to an educational background criterion. Only in two cases, we opted for re-allocation. We chose to seek participants at universities of the third age and in churches; however, there was no predetermined profile of a participant recruited from one or the other. Elderly attending classes at universities of the third age were equally likely to go to church on regular basis. Furthermore, before the assignment, we organized several meetings with lectures about health to ensure informed participation in the study. During the 12 week period, participants from the NW and SG groups were supplemented with a weekly dose of 28,000 IU of vitamin D3, administered orally as droplets. This supplementation treatment did not include any additional supplementation ingredients. Anthropometric data, body composition and blood samples from all subjects were performed at the beginning and the end of the 12 week experiment (Figure 1). In order to counteract any diet-related influence on myokines and other measured proteins, participants were served the same breakfast meal. This procedure was applied on the days when blood samples were taken. The breakfast provided 21.0 g of protein, 21.9 g of fat, 31.8 g of carbohydrates and covered 1/5 of all-day energy demand. The total energy of the mean was equal to 405 kcal.

### 2.2. Ethics Statement

The official approval for the examination was granted by the Bioethical Committee of the Regional Medical Society in Gdansk (KB-26/14), according to the Declaration of Helsinki. Before commencing the study, subjects received a verbal description of the experiment and signed an informed consent form for participation.

### 2.3. Training Protocol

A group consisting of the same research assistants and coaches supervised all training sessions. The 12 weeks of a mesocycle exercise were completed by the NW group (n = 33) in three micro-cycles. Participants had three training sessions per week. Each training session took place 1 h after eating a light breakfast and consisted of three phases: A 10 min warm-up, 45–55 min of Nordic walking and a 10 min cool-down, performed at 60–70% intensity of the maximal heart rate (HR). The intensity of the training was recorded by measuring the Heart Rate (HR) using a Garmin Forerunner 405 with a built-in global positioning system (GPS) Typical, standard Nordic walking poles were used. The main task of the first micro-cycle (six training sessions) was to adapt the body to undertaking regular physical activity and to learn the correct exercise form, mainly the right technique for walking with poles. It also aimed to improve chest mobility and to increase the flexibility of the arms and shoulders. The second micro-cycle included 24 training units and was an essential part of the program, aimed at improving endurance. The training volume (expressed as walking kilometers) was gradually increased, which was inherent in increasing the training intensity. The closing micro-cycle (six training units) was geared towards raising the level of endurance by intensifying the exercise and walking at the fastest possible pace. Standard poles with special NW gloves but no additional telescopes were used for training. Participants did not take part in any other types of exercise throughout the duration of the study; they were also instructed not to change their daily habits (daily activity for example with a grandchild). Training attendance was checked by the instructor; averaging within the range of 85–90%.

### 2.4. Blood Samples 

Blood samples were collected by a qualified nurse from the antecubital vein into vacutainer tubes before and 1 h after the first and last training session in the NW group. On these days, the group received the same breakfast, prepared by the same qualified dietitian. In the SG and CG groups, only fasting samples were taken in the morning at baseline and after the 12 weeks of the study. Serum was separated immediately by centrifugation at 2000× *g* for 10 min at 4 °C and stored at −80 °C until analysis. One portion of each blood sample was separated using centrifuge tubes with aprotinin (catalog no RK-APRO, Phoenix Pharmaceuticals Inc., Burlingame, USA).

The quantification of serum IL-6, High sensitivity of C-Reactive Protein (hsCRP) and BDNF were completed using ELISA kits (R&D Systems, Minneapolis, USA, catalog no. HS600B, DCRP00, DBD00 respectively) according to the manufacturer’s instructions. Intra- and inter-assay coefficients of variability (CVs) were as follows: 7.8% and 9.6% for IL-6, 8.3% and 7.0% for hCRP; 6.2% and 11.3% for BDNF. The detection limit for BDNF was <20 pg mL^−1^. The serum concentration of irisin was determined using competitive enzyme immunoassay kits (Phoenix Pharmaceuticals Inc catalog no EK 067-29) with intra- and inter-assay CVs equal to 4–6% and 8–10% respectively.

The concentration of the vitamin D metabolite, 25-hydroxy D3 (25OHD3), was determined following an Ultra-fast LC/MS/MS procedure with modifications applying a Phenomenex TN-1055. A mass spectrometer SHIMADZU LCMS 8050 HPLC system Nexera X2 column, Agilent Eclipse Plus C18 1.8 μm 2.1 × 100 mm was used for the assay. These methods were described in detail in a previous publication [15].

The concentrations of serum homocysteine, methionine, and cysteine were determined by applying ion-pair reversed phase high-performance liquid chromatography using tandem mass spectrometry IP-RP HPLC-MS/MS (TSQ Vantage Thermo Scientific) [14].

Glucose was measured with an analyzer Cobos 6000, ferritin with a SYSMEX XE 2100, Architect ci 8200 and Test 1 SDL, and iron using the Roche Modular System (Roche Diagnostics, Basel, Switzerland).

### 2.5. Body Composition

Body composition was measured using a multi-frequency plethysmograph body composition analyser (InBody 720, Biospace, Korea) [21]. All data were collected in the morning between 6 and 8 a.m., following an overnight fast.

### 2.6. Statistical Analysis

Statistical analysis was performed using Statistica 12.0 software (Statsoft, Tulsa, Oklahoma). Additionally, figures were made using GraphPad Prism 7. All values were expressed as the mean ± standard deviation (SD). The Shapiro-Wilk test was applied to assess the homogeneity of dispersion from a normal distribution. The Brown-Forsythe test was used to evaluate the homogeneity of variance. For homogenous results, the analysis of variance (ANOVA) for repeated measurements and the post-hoc Tukey test for unequal sample sizes were performed to identify significantly different results. For heterogeneous results, the ANOVA Friedman’s test and the right post-hoc test were applied. To estimate the practical relevance of the ANOVA between group effects and effect sizes (partial eta squared, η_p_^2^) were additionally calculated. The significance level was set at *p* < 0.05. The relationships between the variables were evaluated using the Pearson correlation coefficient.

## 3. Results

### 3.1. Characteristics of Study Participants

All 94 women participating in the project have completed this study without reporting any adverse events. There were not any statistical differences between the age of the groups at baseline as well at the end of the study. The anthropometric characteristics of the female seniors are presented in Table 1. The applied procedure did not induce any significant changes in body composition.

### 3.2. The Effect of A Single Session of Nordic Walking Training 

A single session of NW training resulted in a significant drop of homocysteine. It is worth noting that the decrease of Hcy concentration was mainly observed among those exercising participants with a higher initial concentration of Hcy >10µmol·L^−1^; from 12.4( ± 2.7) to 9.9( ± 2.2) µmol·L^−1^ (*p* = 0.01). Changes among the participants, who were characterized by the concentration of Hcy <10 µmol·L^−1^ were not significant statistically but the tendency was the same 8.3( ± 1.2) to 7.8( ± 2.0) µmol·L^−1^.

**Figure 2:**
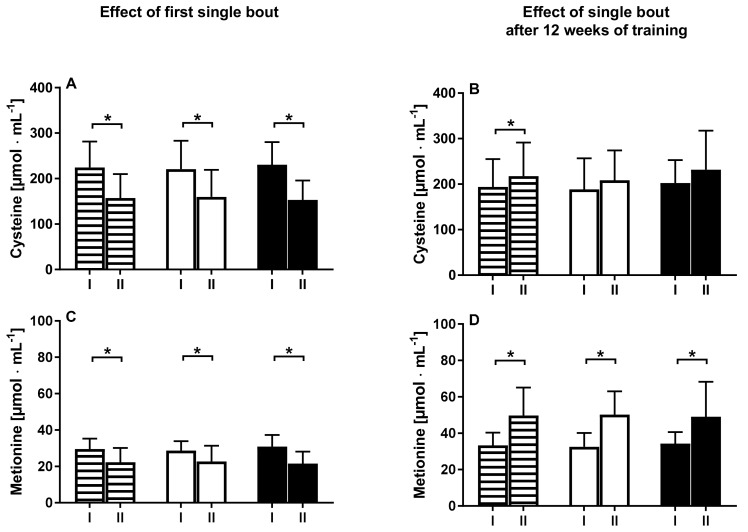
Changes in cysteine and methionine levels between pre (I) and 1 h after (II) exercise for all participants (white-black stripes) and subgroups with a baseline homocysteine level under 10 µmol·L^−1^ (white) and over 10 µmol·L^−1^ (black).

Together, with a drop of Hcy in response to a single session of NW, a decrease of methionine and cysteine was noted (Figure 2).

The drop of these two amino acids was similar between subjects characterized by a high and low concentration of Hcy. A single session of NW training had no effect on the pro-inflammatory protein hsCRP. As expected, a significant rise of myokine IL-6 was noted 1 h after the single session of NW training. Conversely, myokines like BDNF and irisin did not shift in response to the first NW session. Interestingly, a single session of NW exercise performed after 12 weeks of training-induced an increase of IL-6 yet a significant decrease of BDNF. These changes were noted in both groups with low and high Hcy serum concentrations. A single session of NW performed after 12 weeks of training caused the concentrations of methionine and cysteine to increase. This shift was not modified by a baseline Hcy concentration (Table 2).

### 3.3. The Effect of 12 Weeks of Nordic Walking Training on Homocysteine and Iron Status

**Figure 3:**
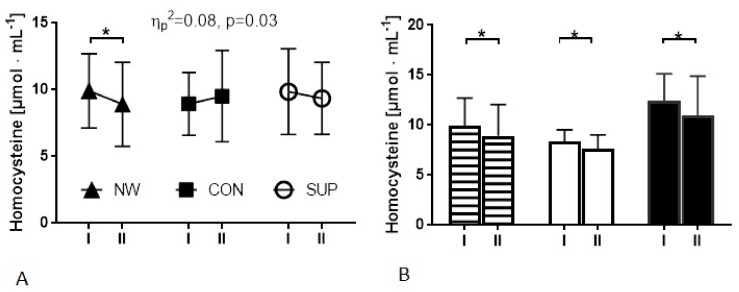
Changes in the resting homocysteine concentration in groups induced by the applied procedure. (**A**) I—at baseline, II—after 12weeks in NW, control and supplemented groups. (**B**) Analysis only in the NW Group; marked columns—all participants from the NW group, white columns—participants from the NW group with a baseline homocysteine ≤10 µmol·L^−1^, black columns—participants from the NW group with a baseline homocysteine >10 µmol·L^−1^, I—at baseline, II—after 12cweeks of NW training.

As expected, in response to the 12 weeks of training, the level of vitamin D was observed to have risen in the supplemented groups (NW and SG). In the CG group, the concentration of vitamin D remained unchanged. The obtained results indicate that the 12 weeks of NW lead to a significant reduction of Hcy. A slight, yet not significant, drop of Hcy was also noted in the SG group, whereas in the CG group the opposite tendency was observed. It is worth noting that changes observed in Hcy were noted in whole NW group, as well as in the subgroups; above and below 10 µmol·L^−11^ was considered (Table 3 and Figure 3). In the NW group, as well as in the SG group, a drop of BDNF was noted. The range of the change magnitude for BDNF was higher in the SG group (55%) compared to the NW group (25%). The 12 weeks of NW training, supported by the vitamin D supplementation, resulted in a decreased in the ferritin level (Table 3). It is worth noting that in the NW group, the lowest level of ferritin was recorded at baseline. Nevertheless, in this group, the changes were the most pronounced. The slight drop was also noted in the SG and CG groups although these alternations were not significant. Although our intervention did not induce significant changes in the resting concentration of glucose (Table 3), we noted an interesting, positive correlation between glucose and ferritin after the whole period of the experiment.

**Figure 4:**
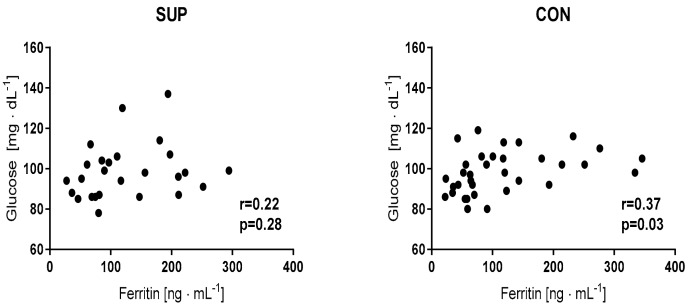
Correlation between glucose and ferritin levels recorded after the 12 weeks of the experiment.

It is of note that, in the NW group after 12 weeks of NW training, the correlation was stronger in comparison to the baseline. A similar tendency also was observed in the SG and CG groups, although the correlations were not so strong (Figure 4).

**Figure 5:**
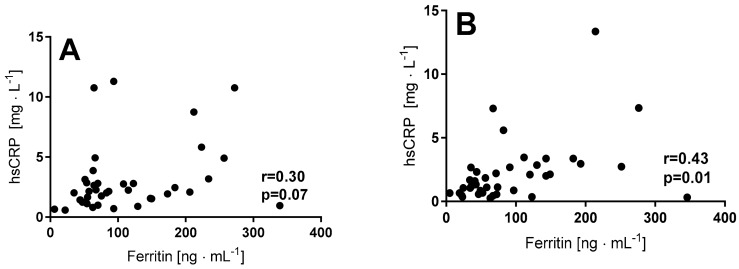
Correlation between ferritin and hsCRP at the baseline and in response to 12 weeks of Nordic Walking training in elderly women in a group with a baseline homocysteine < 10 µmol·L^−1^], (**A**) baseline, (**B**) after 12 weeks of training.

The observed drop in serum ferritin in the NW group was associated with a significant decrease of serum iron, although this tendency depended on the level of Hcy. The decrease of serum iron was more pronounced among those participants characterized by a lower Hcy concentration (<10 µmol·L^−1^). Additionally, there was only a significant correlation between ferritin and hsCRP noted in this group (Figure 5).

The applied procedure of supplementation significantly increased its concentration (Table 3). Based on a previously published study, where the changes induced by training were dependent on the concentration of vitamin D and a cut off of 21 ng·mL^−1^ was considered [22], the same analysis was performed. Overall, the baseline level of vitamin D did not influence the changes in the Hcy concentration but the supplementation had a pronounced impact on its concentration. In both supplemented groups (NW and SG), the level of Hcy decreased and the effect was significant (*p* = 0.05), whereas, in the CG group, the tendency was the opposite—the level of Hcy increased. Multiple pairwise post-hoc *t*-tests revealed significant differences between groups (*p* = 0.01). Furthermore, women from the CG group with a 25(OH)D concentration below 21 ng·mL^−1^ were characterized by a considerably higher level of Hcy compared to those with a 25(OH)D concentration above this cut off concentration.

## 4. Discussion

The main finding of the present study is that 12 weeks of regular NW training supported by supplementation with vitamin D led to a significant reduction of serum Hcy and ferritin. Interestingly, a single session of NW training also induced a significant drop in the serum level of Hcy. There are several studies showing that regular exercise can diminish the serum Hcy level, however, we are not aware of any study where the effect of single exercise has been evaluated. In the case of a single session of exercise, the decrease was more pronounced among those participants characterized by a level of homocysteine above 10 µmol dL^−1^. The reason behind a decrease in the Hcy concentration after a single session of exercise is not known. The plasma methionine concentration has been shown to increase in response to a 40 min moderate intensity session of exercise [23]; this may suggest that exercise somehow stimulates the conversion of Hcy to methionine. In addition, Hcy can be converted to alfa-ketobutyrate and then to succinyl CoA, which is an intermediate of the Krebs cycle [24]. In line with this observation, we have noted that the drop of Hcy recorded in response to a single session of NW was accompanied by a rise of methionine and cysteine after the 12 weeks of the training. Contrariwise, at baseline, a single session of NW exercise led to a drop in both the methionine and cysteine concentrations. These data suggest that the changes in Hcy before and after training have a different biochemical mechanism. Thus, further investigations involving precise diet control are needed to assess whether changes observed after training occurred due to some adaptive changes or dietary modifications.

Six to 12 months of resistance training have been demonstrated to significantly reduce plasma Hcy concentration [2,25]. Our data clearly show that even the 12 weeks of NW training supported with vitamin D supplementation were sufficient to reduce the serum Hcy concentration. It should be noted that the drop in Hcy after the 12 weeks of training was accompanied by a decrease in serum ferritin level. In addition to its ferroxidase activity and role in iron storage, ferritin H has been shown to stimulate folate degradation. Thus, in cells with a high ferritin H protein content, the elevated catabolism of folate has been recorded [10]. Consequently, it is possible that a decrease in body iron stores and ferritin protein induced by regular NW training may have led to an increase the bioavailability of folate, which could be responsible for a decrease in Hcy. 

At the same time, the analysis showed that vitamin D supplementation without exercise was insufficient to diminish the Hcy level. From a large cohort of healthy adults, an inverse relation between 25(OH)D and homocysteine was observed among those with a 25(OH)D concentration below 21 ng·mL^−1^ [22]. In our study, women from the CG group, with a 25(OH)D concentration below 21 ng·mL^−1^, were characterized by a considerably higher level of Hcy compared to those with a 25(OH)D concentration above 21 ng·mL^−1^, which supports the previous observations [22]. The applied supplementation program resulted in a significant growth in the vitamin D concentration. This may explain why we did not find a relation between the decreased homocysteine levels in both NW and SG groups.

The second aim of the study was to evaluate if changes in the pro-inflammatory Hcy concentration were affected by shifts in myokines concentration (irisin and IL-6), which have an anti-inflammatory effect [19]. Additionally, the latest published reviews have emphasized the role of irisin in glucose homeostasis [26] and the regulation of exercise-induced adaptations [27]. Due to the fact that both IL-6 and irisin may affect the BDNF concentration, its level was also determined [20]. In the current study, no relationship between irisin, IL-6 and glucose concentration was noted. Fatouros has observed that a particularly high intensity of exercise induced an increase in the irisin level [27]. Thus, the lack of significant changes to the irisin concentration could have resulted from applying a moderate intensity of NW training. Interestingly, in both of the supplemented groups, a significant drop in the BDNF concentration was noted. This observation corresponds with a previously published study, which linked the supplementation of vitamin D with a decrease in the BDNF concentration [14]. As approximately 70–80% of BDNF originates from the brain, a reduced peripheral concentration may result from its absorption by the structures responsible for maintaining cognitive abilities such as the prefrontal cortex or the hippocampus and also from its uptake by the muscle tissue to support lipid metabolism [28]. Changes in the blood BDNF concentration do not necessarily reflect an increase in BDNF in the brain in response to exercise. They may also signify a reduced BDNF release by the brain or its higher uptake. Most of the data have shown that acute physical training led to an elevation of the peripheral BDNF concentration [29] and the type of training was determined as important for inducing such growth [29]. An increase of BDNF in response to physical training was recorded in healthy subjects [30] as well as among Parkinson’s disease patients [31].

Further research should continue to determine if there are any relationships between irisin and BDNF and metabolic or psychological changes exist in among elderly people. 

Our training program, supported by the vitamin D supplementation, has significantly reduced the level of the pro-inflammatory serum homocysteine and these data are in agreement with previous observations by Gmiat, who noted that the same schedule of the training procedure reduced the level of the pro-inflammatory protein—HMGB1 and this reduction being negatively correlated with irisin concentration [14]. Although in the present study no changes in irisin were recorded, a slight drop in the high-sensitivity C-reactive protein was noted. At the same time, both coupling supplementation and training and even applying supplementation alone caused changes in the serum ferritin. The lower the level of ferritin registered, the lower the level of glucose was observed, which may suggest that the applied procedure improved glucose uptake.

Our study has some limitations, which pose questions that should be addressed in future investigations. We would like to emphasize that we did not ask our subjects to train without receiving vitamin D supplementation. Working with subjects who underwent training but did not receive supplementation would have been unethical given the common vitamin D deficiency among people in this age group. Although there were no differences in education level between groups, we cannot exclude that the recruitment places (churches and Universities of Third Age) could have had an impact on the results obtained. Thus, uniform recruitment should be considered in further investigations.

## 5. Conclusions

Overall, our study has shown that coupling physical training with the supplementation of vitamin D decreased the homocysteine and ferritin levels in elderly women. Although the NW group had the lowest ferritin level at the baseline, in this group a range of changes were the most significant. Therefore the obtained effect supports the meaning of regular training. Moreover, this observation reinforces the earlier observation of interconnection between iron and homocysteine metabolism.

## Figures and Tables

**Table 1:** Characteristics of the participants before and after the applied procedure.

	Nordic Walking			Supplemented Group			Control Group		Baseline	
	Baseline	After 12w	*p*	Baseline	After 12w	*p*	Baseline	After 12w	*p*	*p*
Age (years)	67.8 ± 5.4			66.9 ± 6.2			68.2 ± 6.7			0.71
Body-Weight (kg)	68.7 ± 9.8	69.2 ± 9.1	0.11	69.2 ± 10.1	70.3 ± 10	0.00	72.4 ± 12.1	73.2 ± 11.8	0.48	0.34
BMI (kg m^−2^)	26.3 ± 3.9	26.5 ± 3.7	0.06	26.4 ± 3.5	26.8 ± 3.7	0.20	27.4 ± 3.9	27.9 ± 4.1	0.10	0.43
Fat (kg)	23.9 ± 7.4	24.4 ± 7.5	0.35	25.1 ± 7.3	25.9 ± 7.7	0.11	27.2 ± 7.9	27.6 ± 8.3	0.75	0.21
Fat (%)	34.2 ± 6.4	34.7 ± 7.6	0.37	35.5 ± 6.7	36.1 ± 7.1	0.33	36.7 ± 6.7	35.7 ± 8.6	0.37	0.28
TBW (kg)	32.8 ± 2.9	32.9 ± 3.7	0.91	32.5 ± 3.7	32.7 ± 3.8	0.90	33.1 ± 4.9	33.5 ± 4.8	0.33	0.81
FFM (kg)	44.7 ± 4	44.8 ± 5	0.93	44.2 ± 5	44.6 ± 5.1	0.87	45.1 ± 6.6	45.6 ± 6.5	0.41	0.80

Values are means (±SD); 12w—12 weeks, baseline—differences between groups at baseline, BMI—body mass index, Fat—fat mass, Fat%—percentage of body fat, TBW—total body water, FFM—free fat mass, all participants n = 94; NW group n = 33; SG group n = 27, CG group n = 34.

**Table 2:** The effect of the first and last single training session of Nordic Walking on immunological response.

Variable	Before Training	1 h after Training	*p* Value	Confidence Interval	
				−95%	+95
Homocysteine (µmol·dL^−1^)	9.91 (2.78)	8.70 (2.31)	0.01 *	−2.16	−0.27
hsCRP (mg·L^−1^)	2.60 (2.21)	2.50 (1.80)	0.53	−0.67	0.38
IL-6 (pg·mL^−1^)	1.32 (0.68)	2.19 (1.29)	0.01 *	0.19	1.43
BDNF (ng·mL^−1^)	42.74 (19.92)	44.43 (18.81)	0.96	−8.68	10.00
Irisin (ng·mL^−1^)	12.00 (4.44)	12.66 (4.68)	0.46	−1.14	2.47
**After 12 weeks of NW training**					
Homocysteine (µmol·dL^−1^)	8.90 (3.14)	9.17 (2.97)	0.46	−0.97	0.45
hsCRP (mg·L^−1^)	2.82 (3.04)	2.45 (6.31)	0.34	−1.15	0.41
IL-6 (pg·mL^−1^)	1.59 (0.78)	2.26 (1.02)	0.00 *	0.43	0.94
BDNF (ng·mL^−1^)	31.93 (15.91)	26.16 (13.03)	0.04 *	−12.65	−0.1
Irisin (ng·mL^−1^)	12.45 (6.31)	12.84 (5.29)	0.83	−1.37	1.47

Values are means (SD); IL-6—interleukin 6, BDNF—brain-derived neurotrophic factor, hsCRP—High sensitivity of C-Reactive Protein, *—Significantly different to baseline (resting condition), confidence interval calculated for range of change.

**Table 3:** Changes induced by the applied procedure in the NW group—training and supplementation, in SP by supplementation and values of control group (CG).

	Nordic Walking (n = 33)		Control Group (n = 34)		Supplemented Group (n = 27)		rANOVA	
	Baseline	After 12w	Baseline	After 12w	Baseline	After 12w	Interaction	η_p_^2^
Homocysteine (µmol·L^−1^)	9.91 (2.78)	8.90 * (3.14)	8.93 (2.35)	9.51 (3.42)	9.85 (3.20)	9.35 (2.69)	*p* = 0.03	0.08
Ferritin (ng·mL^−1^)	94.23 (62.49)	73.15* (47.04)	127.94 (84.94)	117.05 (87.51)	137.85 (81.88)	124.68 (72.11)	*p* = 0.29	0.03
Glucose (mg·dL^−1^)	96.64 (12.25)	95.06 (12.05)	101.88 (12.33)	98.29 (10.47)	99.69 (13.62)	98.54 (13.64)	*p* = 0.33	0.01
Vitamin D (ng·mL^−1^)	23.01 (9.97)	59.48* (27.61)	24.64 (11.61)	22.11 (9.52)	27.37 (8.14)	63.29 * (14.25)	*p* = 0.00	0.51
BDNF (ng·mL^−1^)	42.74 (19.92)	31.93 * (15.91)	33.52 (12.61)	28.96 (12.86)	37.75 (8.08)	16.94 * (12.78)	*p* = 0.01	0.11
Irisin (ng·mL^−1^)	12.00 (4.44)	12.45 (6.31)	11.66 (4.06)	11.89 (3.71)	12.17 (7.62)	11.30 (6.51)	*p* = 0.49	0.02
IL-6 (pg·mL^−1^)	1.32 (0.68)	1.59 (0.78)	1.89 (0.98)	1.86 (1.56)	2.66 (1.38)	3.11 (1.92)	*p* = 0.56	0.01
hsCRP (mg·L^−1^)	2.60 (2.21)	2.82 (3.04)	3.73 (3.31)	2.75 (3.18)	2.01 (1.92)	2.15 (1.97)	*p* = 0.08	0.06

Values are means (SD). Interaction—Group x time interaction, partial eta squared (ηp2) values are provided to estimate the effect sizes of the repeated measurment analyses of variance (rANOVAs). *—Significant differences after 12 weeks, *p* < 0.05, 12w—12 weeks.
